# Pancreaticogastrostomy with a lumen-apposing metal stent for retrieval of a proximally migrated pancreatic stent after Whipple surgery

**DOI:** 10.1055/a-2930-9040

**Published:** 2026-08-18

**Authors:** Delfien Coevoet, Pierre H. Deprez

**Affiliations:** 1 Department of Hepatogastroenterology 70492 Cliniques universitaires Saint-LucBrusselsBelgium; 2 Department of Hepatogastroenterology 83415 Université catholique de LouvainLouvain-la-NeuveBelgium

Migration of pancreatic stents is an uncommon complication (approximately 5%) of pancreatic duct (PD) stenting, potentially causing PD injury. Various techniques have been described for endoscopic retrograde retrieval. 1​ However, retrograde access is not always feasible due to PD stenosis or postsurgical anatomical alterations. 2​ 3​ ​4​

**Fig. 1:**
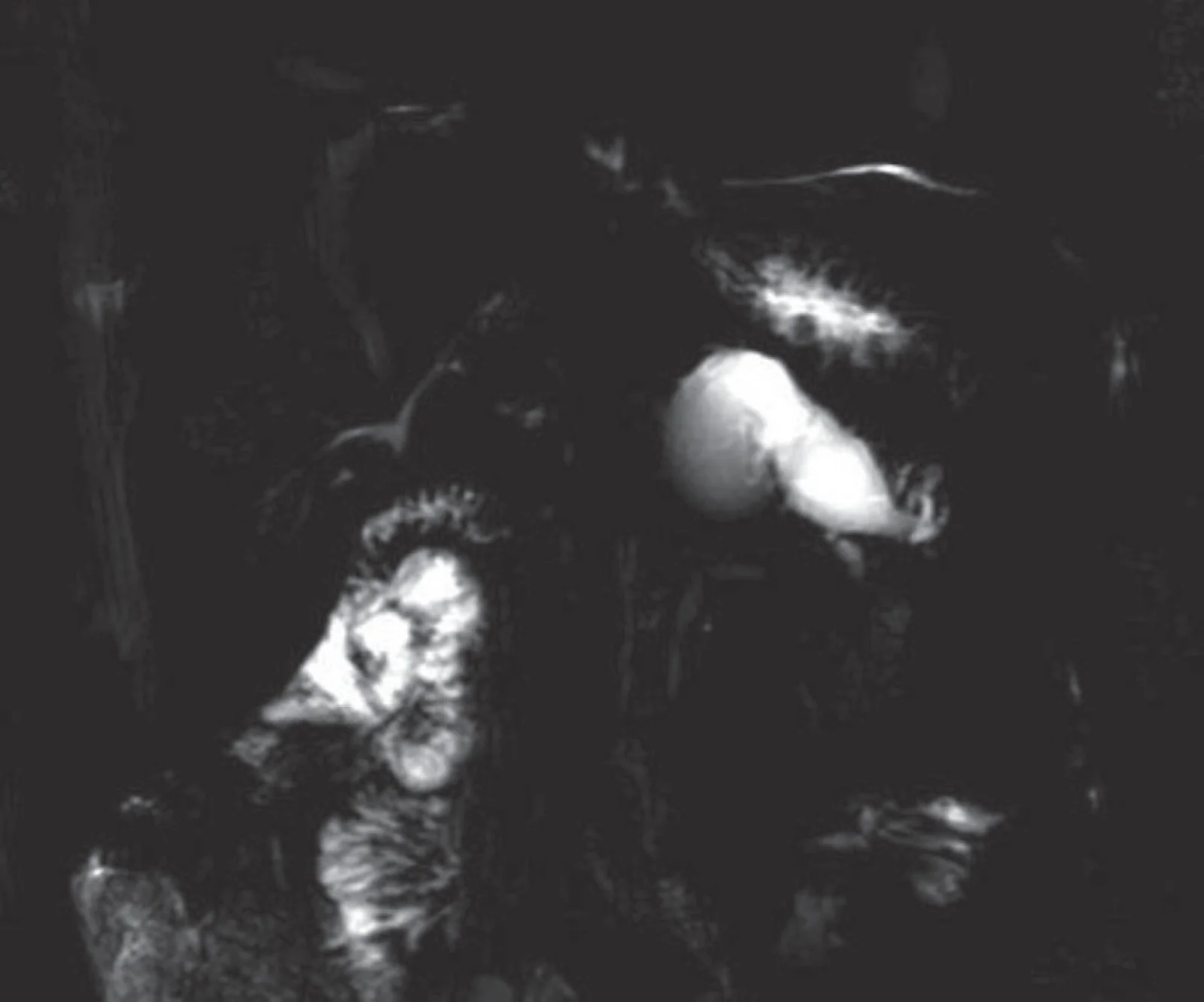
Pancreatic duct dilatation up to 35 mm.

**Fig. 2:**
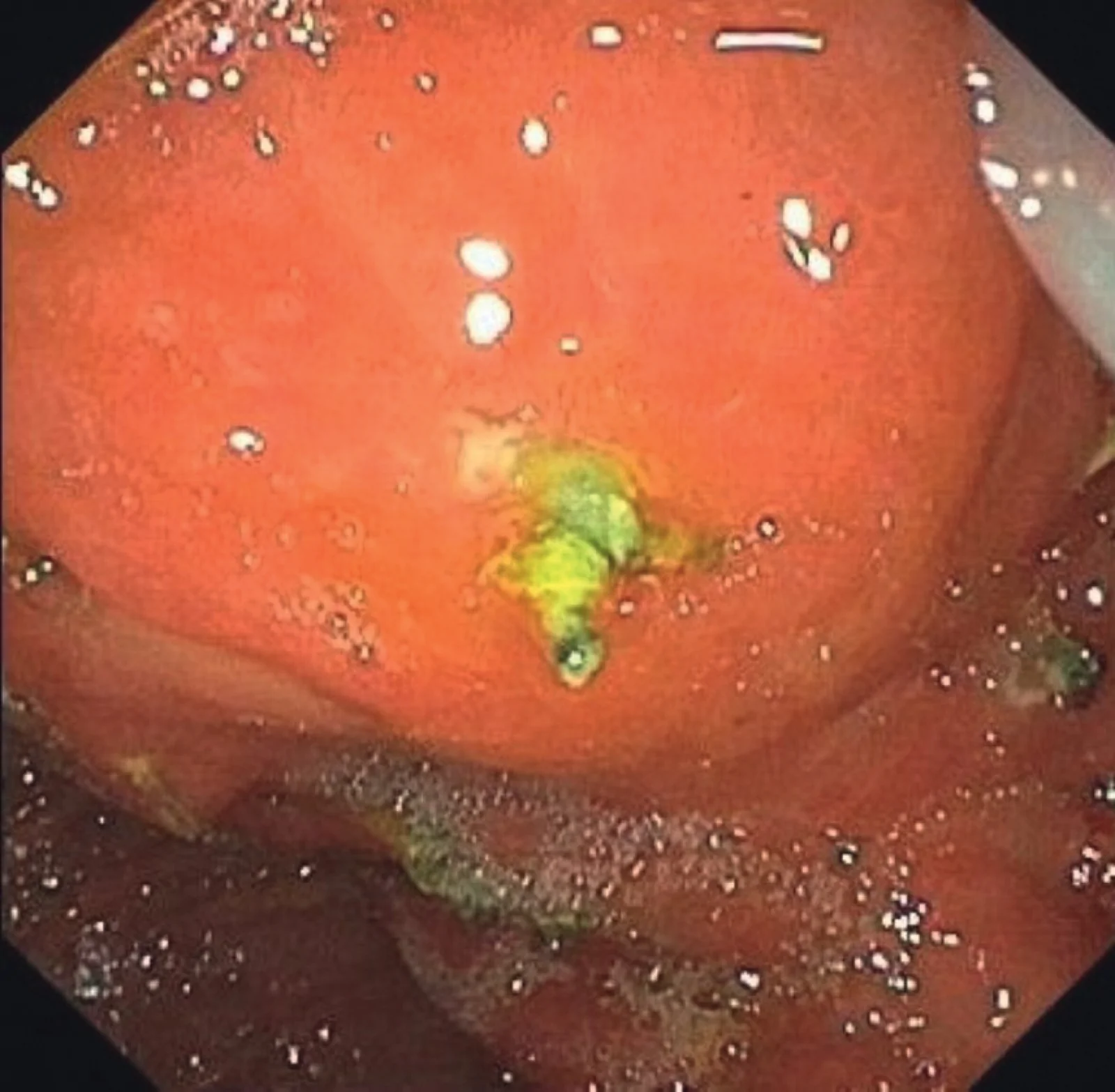
Pancreaticogastric anastomotic strictures.

A 32-year-old woman with a history of a duodenopancreatectomy with pancreaticogastric anastomosis, performed 11 years earlier for a solid pseudopapillary pancreatic neoplasm, was referred with progressive PD dilatation. She presented with epigastric pain and elevated serum lipase levels. Magnetic resonance imaging demonstrated PD dilatation from 17 to 35 mm ( Fig. 1 ). Endoscopic retrograde cholangiopancreatography, performed in October 2023, revealed a stricture at the surgical anastomosis with no possible guidewire passage ( Fig. 2 ).

Subsequent endoscopic ultrasound (EUS) and fluoroscopic evaluation confirmed pancreatic atrophy, a markedly dilated PD and an intrapancreatic migrated surgical stent.

**Fig. 3:**
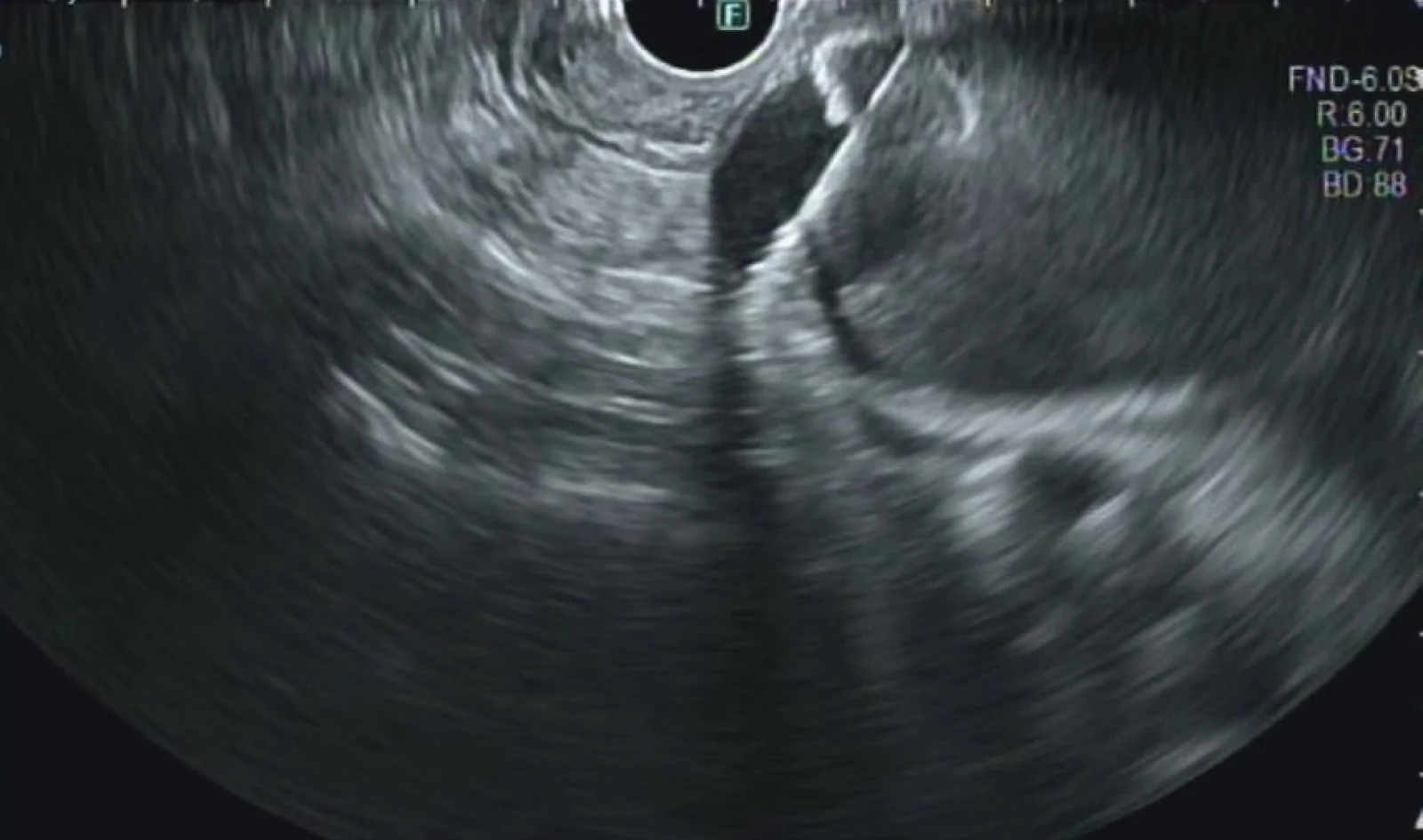
Placement of a LAMS in the dilated pancreatic duct from the stomach.

To facilitate PD access for stent retrieval, an EUS-guided pancreaticogastrostomy was performed using a lumen-apposing metal stent (LAMS, 6×8 mm; **Figs.** 3 **,** 4 , and Video 1 ). Immediate self-limiting bleeding occurred, which was treated with peristomal adrenaline injection, without further complications.

**Fig. 4:**
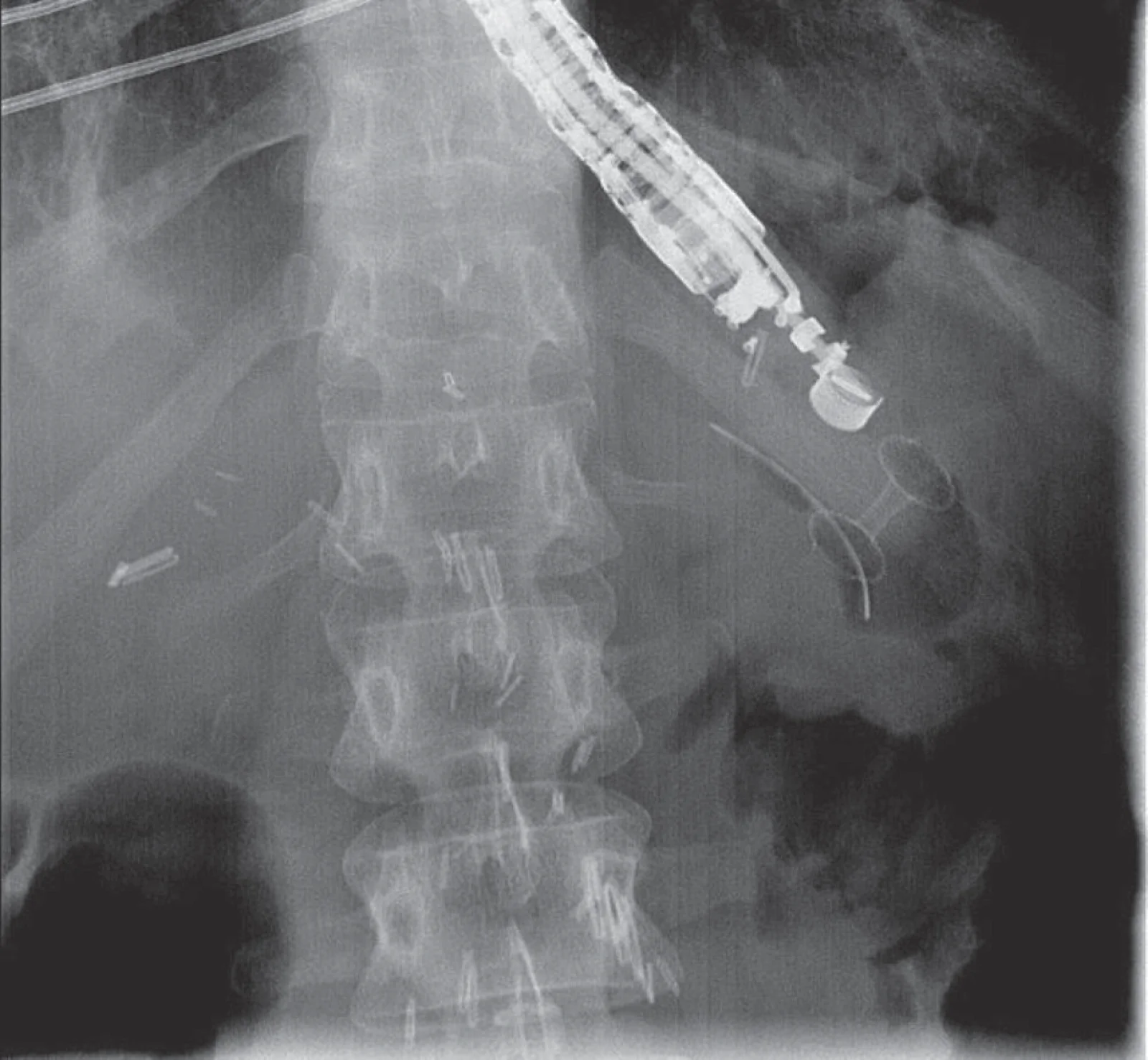
LAMS placed to retrieve the migrated pancreatic stent during a second procedure.

**Video 1** LAMS (6×8 mm) placement in a dilated pancreatic duct creating a pancreaticogastrostomy. Eight weeks later, the migrated stent was retrieved using rat-tooth forceps through the LAMS tract.


**Fig. 5:**
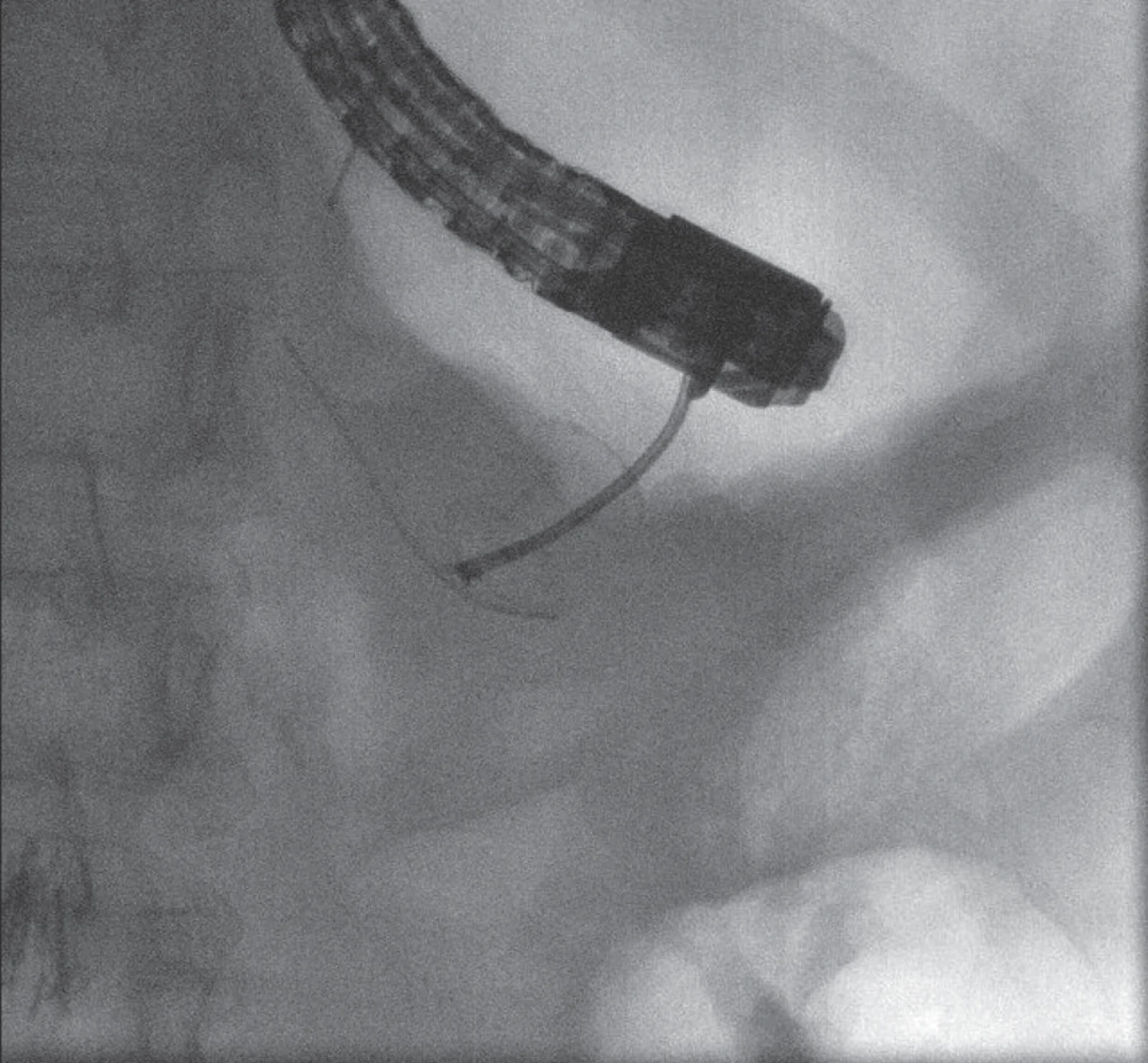
Retrieval of the migrated pancreatic stent using a rat-tooth forceps trough the LAMS.

Two months later, the migrated stent was successfully retrieved using a rat-tooth forceps introduced through the LAMS-created pancreaticogastrostomy tract. Following uneventful stent retrieval ( Fig. 5 ), the fistulous tract was cannulated with a ball-tip catheter and attempts were made to advance the guidewire antegrade across the anastomotic stricture, but passage could not be achieved. A plastic stent (single-pigtail, 6cm 5Fr) was placed to maintain the fistula tract open. No post-procedural complications occurred. Three months later, CT showed the regression of the PD diameter to 10 mm and the patient remains asymptomatic up till now.

To our knowledge, this is the first report of a LAMS placement within the PD to facilitate the retrieval of a migrated pancreatic stent. This case highlights an effective EUS-guided method that may obviate the need for surgical intervention in altered anatomy.

Endoscopy_UCTN_Code_TTT_1AS_2AI
